# Q fever in urban area – an emerging zoonosis

**DOI:** 10.1186/1471-2334-14-S7-P86

**Published:** 2014-10-15

**Authors:** Cristina Popescu, Alina Lobodan, Raluca Dulamă, Anca-Ruxandra Negru, Mihaela Rădulescu, Cătălin Tilişcan, Gabriel Adrian Popescu, Viorica Poghirc, Raluca Popescu, Georgiana Jugănaru, Victoria Aramă

**Affiliations:** 1 National Institute for Infectious Diseases "Prof. Dr. Matei Balş", Bucharest, Romania; 2 Carol Davila University of Medicine and Pharmacy, Bucharest, Romania

## Background

Q fever is a zoonosis with reported outbreaks in rural areas, related to farms and farm animals. In the urban area, the source of infection is almost always unknown and can be related to windborne spread of *Coxiella burnetii*.

Objective: To emphasize the importance of *Coxiella burnetii* etiology in prolonged febrile syndrome. We want to point out that Q fever can become a real threat even in the urban area.

## Methods

We made a retrospective analysis of Q fever cases treated in Matei Balş Institute between August 2011 and July 2014, insisting on the delay of diagnosis and treatment in these cases. The diagnosis was made using ELISA for IgM antibodies against *Coxiella* and the confirmation was made by immunofluorescence assay (phase I and phase II antibodies).

## Results

Eighty-nine patients with a mean age of 49.7 year-old and a sex ratio M:F = 1.6:1 were included: 9 patients in 2011 (10.11%), 21 in 2012 (23.59%), 33 in 2013 (37.07%) and 26 in the first semester of 2014 (29.21%). An ascendant trend was observed. Almost all cases were registered in the warm season (from May to September) - 83.14%. In the urban area were recorded 84.26% cases. In only 5 cases, the patients were at risk of contamination. The organs affected during *Coxiella* infection were: lungs (82.02%), liver (76.04%) and heart (6.74% - 2 cases of myocarditis and 4 cases of endocarditis). The most common clinical presentation was for acute febrile disease associated with: pneumonia and hepatitis – 55.05%, pneumonia only – 23.59%, hepatitis only – 14.06%, endocarditis plus pneumonia and hepatitis – 4.49%, myocarditis plus hepatitis – 2.24%. More than two SIRS criteria were found in 70.7%; a procalcitonin level >2 ng/mL was found in 18.66% of cases. The delay between the first sign of disease and the diagnosis varied between 2 and 60 days with a mean duration of 15.21 days. The mean delay until an active antimicrobial was administered was 8.03 days. Doxycycline was the most utilized antimicrobial – 84.26%. In 15.74% of cases, fluoroquinolones were administered. The mean duration of therapy in non-endocarditis patients was 15.5 days.

## Conclusion

*Coxiella burnetii* infection should be considered in patients with prolonged fever of unknown etiology. The association between pneumonia and hepatitis in a case of prolonged febrile syndrome is highly suggestive for Q fever even though the patient lives in urban areas.

